# Seroprevalence of Influenza A Virus in Dromedaries in North-Western Nigeria

**DOI:** 10.3390/pathogens11121476

**Published:** 2022-12-05

**Authors:** Andrew M. Adamu, Morgan Furlong, Samson Ogunlade, Alex A. Adikwu, Annabel S. Anyang, Arhyel Malgwi, Adeiza M. Abdulrahman, Nma A. Bida, Olajide A. Owolodun, Oyelola A. Adegboye

**Affiliations:** 1 Australian Institute of Tropical Health and Medicine, Building 48, James Cook University, Townsville, QLD 4811, Australia; 2 College of Public Health, Medical and Veterinary Sciences, James Cook University, Townsville, QLD 4811, Australia; 3 Department of Veterinary Public Health and Preventive Medicine, University of Abuja, Abuja 900105, Nigeria; 4 Department of Veterinary Public Health and Preventive Medicine, College of Veterinary Medicine, Federal University of Agriculture, Makurdi 970101, Nigeria; 5 Nigeria Field Epidemiology and Laboratory Training Program, African Field Epidemiology Network, Abuja 900105, Nigeria; 6 Biotechnology Division, National Veterinary Research Institute, Vom 930001, Nigeria; 7 Public Health and Tropical Medicine, College of Public Health, Medical and Veterinary Sciences, James Cook University, Townsville, QLD 4811, Australia; 8 World Health Organization Collaborating Center for Vector-Borne and Neglected Tropical Diseases, College of Public Health, Medical and Veterinary Sciences, James Cook University, Townsville, QLD 4811, Australia

**Keywords:** influenza A virus, camels, dromedaries, Nigeria, One Health, zoonosis, environmental contamination

## Abstract

Although influenza A virus is endemic in wild waterfowl, domestic poultry, swine, humans, bats, cetaceans, dogs, and horses, there is a paucity of data on the potential role of camels in zoonotic transmission of the virus. To estimate the seroprevalence of the influenza A virus in camel populations, four local government areas of Nigeria that share an international border with the Niger Republic were selected. Blood samples from 184 one-hump camels (dromedaries) were collected and tested for influenza IgG antigen by ELISA. Each camel’s demographic variable, such as age, gender, location, production system, and usage, was recorded. The overall seroprevalence rate of influenza virus IgG in this study was 10.33% (95%CI: 6.33–15.66%). In the bivariate model, there was no significant difference in gender, age, site location and production system, except for usage. There was a significantly lower seroprevalence rate among camels used for labour (odds ratio (OR) = 0.34, 95% CI: 0.10–0.97) than those used for meat consumption; however, not after adjusting for other variables in the model. Increase surveillance through early detection, prediction, and risk assessment of pathogens in animal reservoirs and environmental contamination as One Health strategies to reduce potential human spillover is recommended. Molecular epidemiology studies could better elucidate the role of camels in the dynamics of disease transmission pathways.

## 1. Introduction

Influenza A viruses (IAVs) belong to the family *Orthomyxoviridae*, and contain single-stranded RNA genomes of eight gene segments [1,2]. IAVs are further classified into subtypes based on variations of their envelope glycoproteins: hemagglutinin antigen (HA) and neuraminidase antigen (NA) [3]. There is a vast diversity of IAV subtypes in wild waterbirds, their natural host, comprising 16 HA and 9 NA different subtypes. Some IAV subtypes have also established endemic circulation in a range of animal species, including domestic poultry, swine, humans, dogs, horses, rodents, lagomorphs, anteaters, cetaceans, non-human primates, and bats. Cross-species transmission of IAVs into humans has led to four influenza pandemics in the last ~105 years [4].

Studies have detected IAVs in wild and domestic animals throughout Africa [1,5,6]. IAV subtypes H1N1, H3N8, and H5N1 have been detected in dogs and cats from Nigeria, Kenya, and Egypt [1]. Highly pathogenic avian influenza (HPAI) A/H5N1 outbreaks have been reported in numerous African countries, including Nigeria, where an estimated 1.6 million poultry deaths have been reported since 2015, specifically in live bird markets near Lagos [6,7]. Wild bird surveillance in southern Africa has also detected a wide range of IAVs, including viruses from H1, H3, H5, H6, and H7 subtypes. Recent studies have suggested that one-humped camels (dromedaries) may be a host and a potential zoonotic source of IAVs [8,9]. This study aimed to estimate the seroprevalence of influenza A virus antibodies and associated risk factors amongst dromedary camels in the northwestern region of Nigeria. 

## 2. Materials and Methods

### 2.1. Study Area

**Figure 1:**
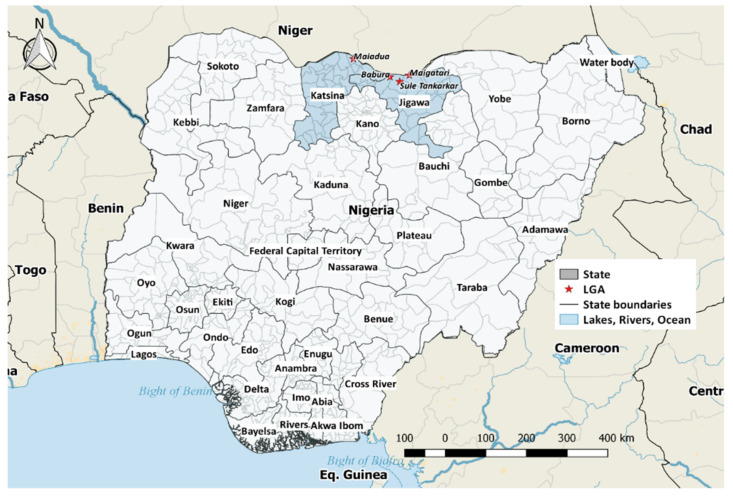
Map of Nigeria showing the study area. Katsina State and Jigawa State are shaded with four LGAs Babura, Maiadua, Maigatari, and Sule Tankarkar indicated with stars.

The study was conducted in the northwest geopolitical zone of Nigeria. Four LGAs were selected for the study based on the relatively high camel population. Two of these local government areas (Maigatari and Mai’adua) have international livestock markets where diverse livestock such as camels, cattle, horses, donkeys, sheep, and goats are traded. Depending on the animal species, some of them originate from Niger Republic, Mali, Chad, and Cameroon. They are purchased from these northern markets and transported to the southern part of Nigeria, Warri (Delta state), Port Harcourt (Rivers state), Lagos, and Abuja, while some of them go all the way to Ghana. These local government areas (Maigatari, Babura, and Suletankarkar) are found in Jigawa state, while Mai’aduwa is a local government in Katsina state (Figure 1). All selected LGAs share an international border with parts of the Republic of Niger. Livestock are transported into these LGAs on foot through the porous borders. The average mean temperature of the study areas is 34 °C, with two distinct seasons (rainy and dry seasons) in the Sahel savannah. Most inhabitants of these LGAs are either cash crop farmers, livestock herders, or hunters

### 2.2. Study Design

A cross-sectional study was conducted with a simple random method on apparently healthy camels from Jigawa and Katsina State. Although based on Thrusfield’s formula [10], assuming an expected prevalence of *p* = 8.3% from previous studies [9], a 95% level of confidence interval (z), and 5% precession (d), the estimated sample size (*n*) was 117, we sampled 184 camels to improve the precision of the estimation.

### 2.3. Blood Sample Collection

For blood samples to be collected, camels were properly restrained in a crouching position. Five millilitres (5 mLs) of blood were collected from each animal using a 10 mL syringe and an 18G needle. The blood was gently transferred into a non-anticoagulant (plain) sample bottle and labelled appropriately. The labelled bottles were kept in a slanting position in a cooler on ice packs and were transported within 24 h to the Viral Research Division of the National Veterinary Research Institute Vom, Plateau State, where they were centrifuged at 10,000× *g* for 5 min to allow for proper separation of serum from the clotted blood. Using a sterile pasture pipette, the serum was extracted into 2 mL cryovial tubes, which were appropriately labelled and stored at −80 °C until used. 

### 2.4. Cross-Sectional Survey

In addition to the blood samples, a cross-sectional survey was conducted to collect information on animal demographics, location, living conditions, and usage. The demographic variables were sex, male (bull) or female (cow), age in years, and location of the four LGAs. Living conditions variables include living areas (fenced or not-fenced), production system (extensive or semi-intensive), water sources (stream, well or borehole), veterinary access (yes or no), and usage (meat, milk, labour). For the usage, farmers were asked if their herds were for meat or milk consumption or labour.

### 2.5. Laboratory Analysis

A competitive enzyme-linked immunosorbent assay kit (c-ELISA) was performed as previously described [11] using commercially available ID Screen^®^ influenza A antibody competition multi-species excellent specificity and sensitivity of over 95% (ID.vet, Grabels, France). The sera were screened for the detection of antibodies against the nucleoproteins of the influenza A virus in camels. Briefly, the c-ELISA was performed in two 96-well antigen-coated microplates. Ninety microliters (90 µL) serum along with test volumes were used in each well of the test assay. The incubation of the antigen-coated microplate was performed for 1 h at 37 °C. The wells were emptied and washed five times with 300 uL of the wash solution, drying of wells was avoided between each wash. Then, 100 uL of the antibody-peroxidase conjugate was added to each well, and the plates were further incubated for 30 min at 21 °C. The plates were then washed three times, and 50 uL of substrate solution was added to each well and incubated for 10 min at 21 °C in the dark. The reaction was stopped by adding 50 uL of the stop solution to each well. The optical density (OD) measurement was read using an ELISA reader spectrophotometer (Thermoscientific™ Multiskan™ MA, USA) set at 450 nm. The competition percentage (S/P%) was calculated as the ratio of the optical density to the calculated mean of the positive control multiplied by 100. Which was compared with the positive and negative controls of each microplate reader according to the manufacturer’s guide. The sample was considered seropositive when the test produced an S/P% ≤ 45%, while serum samples between 45% and 50% were considered equivocal, and samples ≥50% were considered negative.

### 2.6. Data Analysis

The data were presented as frequencies and percentages with a Clopper–Pearson 95% confidence interval (CI) for prevalence. The seropositivity was compared to animal characteristics and husbandry practices using Chi-square tests. Firth logistic regression [12,13] was used to investigate the associations between risk factors and seropositivity. We used Firth’s approach because it is more appropriate for rare events, provides bias-reduction for small sample sizes, and yields finite and consistent estimates in case of separation. The analysis was implemented in R version 4.0.1, and the inference was based on a 5% significance level. Quantum Geographic Information System (QGIS) software version 2.18 was used to map seroprevalence in the study region.

## 3. Results

Table 1 describes the 184 camels sampled from Babura, Maiadua, Maigatari, and Sule Tankarkar local government areas (LGAs) in Nigeria. The median (IQR) age of the dromedaries was 9 (range: 5–12) years, and 54.89% (101) were males. Most of the animals drink from the stream (*n* = 163; 88.59%), while a proportion of them was used for labour (*n* = 80; 43.48%). The serological survey revealed most farmers practised extensive production systems (*n* = 158; 85.87%). Of the 184 sera tested, 19 (10.33%, 95% CI: 6.33–15.66%) were positive for influenza A antigen by ELISA (Table 1). Although male camels (bulls) were found to have higher seroprevalence, 14 (13.86%, 95% CI: 7.79–22.16%) than female camels (cows), 5 (6.05%, 95% CI: 1.98–13.5%), this was not statistically significant (*p* = 0.086).

Seroprevalence only differed significantly based on camel usage in the univariate analysis (Table 1). The likelihood of seropositivity was significantly lower in camels used for labour (odds ratio (OR) = 0.34, 95% CI: 0.10–0.97) compared to those used for meat and milk. There was no significant difference in the seroprevalence between male and female camels, between old and young camels, among LGAs, production systems, living areas, veterinary access, or water source. After adjusting for all variables, none of the animal characteristics was associated with seropositivity. 

## 4. Discussion

This study indicates the presence of antibodies to influenza A virus in dromedaries in Nigeria. An overall seroprevalence of 10.3% was reported from the four studied areas in two northern states that share an international border with the Niger Republic. Nigeria’s camel population comprises those bred in Nigeria and imported camels from neighbouring countries (Niger and Chad). Some camels from further afield countries such as Sudan, Ethiopia, Burkina Faso, and Mali also find their way into Nigeria [14], especially during the dry season. There are few studies on influenza A virus in camels in Nigeria; Olaleye et al. [15] reported a seroprevalence of 0.6% in Borno State, while Chu et al. [8] reported 8.3%. The seroprevalence rate for influenza A virus antibodies in this study compared to previous studies [9,15] in Nigeria indicates an increasing trend in the occurrence of influenza A in camels in Nigeria. This may be due to the increased population of camels and increased human contact with camels [16]. Similarly, the seroprevalence rate observed in this study is higher than the 1.7% reported in Saudi Arabia [8] and 4.7% in Sudan [17]. 

Chu et al. [9] observed that the H1N1 (pdm09) influenza A virus detected in camels from their study was the same strain that was circulating in humans as it coincided with influenza season in Nigeria, suggesting the possibility of reverse zoonoses. Camel samples in this study were collected from the field, contrary to Alghamdi et al. [7], who sampled from the abattoir. There is a possibility that camels could be exposed to influenza A viruses as they travel long distances across arid and semi-arid areas [14], thereby being exposed to influenza viruses when encroaching upon migratory bird sanctuaries, such as Yusufari and Dagona, Yobe state, Nigeria [18]. These birds travel from different parts of the world (North America, England, Germany, Netherlands, Italy, and Israel) from October to March during their annual migrations [18]. The wetlands play a significant role in the livelihood of nomadic pastoralists who take their livestock to these oases to drink water during this period. Therefore, increasing the interactions between these animals and the avian species that have converged. 

Camels and camel products such as meat and milk have gained acceptance with increasing population and climate change. Camels used for labour purposes were found to be statistically associated with lower seroprevalence compared to those used for meat. This could be a concern if camels could be established as reservoirs of influenza A virus, as consumption of their products is common in northern Nigeria among camel pastoralists [16]. Based on product type, camels from an extensive system had higher seropositivity than an intensive system. Most camels in northern Nigeria are raised on an extensive production system where they can move to different terrains to access the feed, which may increase the opportunities for exposure to wetlands contaminated with influenza A viruses by migrating waterbirds. 

Conversely, intensive rearing systems can provide ideal conditions for the rapid spread of respiratory viruses such as influenza once they have been introduced [15]. The study areas are contiguous to the Hadejia-Jama’are-Nguru wetlands, where migratory birds perch. Interaction between camels and migratory birds occurs at drinking points along the plains; this may result in the “mixing” of influenza viruses. Should this happen, novel strains may evolve and circulate in indigenous populations.

In this study, bulls had higher seroprevalence than cows, although there was not a statistically significant difference between sex. Older camels greater than five years had a higher prevalence than younger camels. Similarly, free-roaming camels recorded higher seropositivity than fenced camels. This can be attributed to increased activities among both older camels and free-roaming camels, such as travelling long distances in search of feed and interacting with diverse animal species, both wild and domestic, as they cross boundaries into new ecological niches. This is not surprising because, during their movement, most of these camels have access to water from the streams rather than well water, as observed in this study. Among study areas, camels from Sule Tankarkar local government areas recorded high seropositivity and were four times more likely to be infected with influenza A virus than other local government areas. This local government area has two transhumance routes that link to other parts of the Jigawa state grazing routes [14,15]. Livestock from other African countries interacts with one another in this place, creating another complex transmission pathway for not only influenza A virus but also introducing other zoonotic emerging and re-emerging infectious disease pathogens, such as Rift Valley fever virus [14] and hepatitis E virus [16]. 

A seasonal variation investigation (dry season versus rainy season) study, which can assist in comparing seasonality, could not be carried out in this study. This study investigated a larger sample size than previous studies [9,15]. The data suggest the presence of antibodies to influenza A virus in camels, and age, sex, and location were revealed as potential risk factors associated with seropositivity. There is a need to increase surveillance of influenza A virus in camel populations in Nigeria and Africa. Early detection, prediction, and risk assessment of pathogens in animal reservoirs and environmental contamination as One Health strategies could reduce potential human spillover. Thus, this study recommends the following One-Health strategy, (1) a joint veterinary, medical, and environmental scientist to work on investigative research on camels, humans, and other animal interactions, including avian species. Human surveillance should target “influenza-like illness (ILI)” in sentinel sites as a means of an early detection system, (2) environment scientists and virologists should form teams to collect fecal samples polluting the environment and water bodies.

In summary, this confirms that influenza A virus circulates in camels in Nigeria. Samples were collected from border communities between Nigeria and Niger Republic. There is cross-border movement of camels and other animals, with an imminent possibility of infected camels disseminating viruses among susceptible animal hosts either in market settings or at watering points. Zoonosis is also possible as these animals could easily transmit live viruses to their human owners due to close associations. Influenza A viruses are zoonotic agents; camel herders in these regions associate very closely with their animals. The overarching impact of this association is the maintenance and circulation of influenza between animals and humans in border settlements. Further studies are needed to determine the molecular epidemiology of influenza A viruses in African camels to determine if transmission to this species is through reverse zoonoses (human–camel) or through exposure to avian influenza viruses from migrating waterbirds.

## Figures and Tables

**Table 1:** Comparison of influenza A antibodies and baseline characteristics of camels.

	*n* (%)	Positive, *n* (%, 95% CI)	OR (95% CI)	*p*-Value	aOR (95% CI)	*p*-Value
Overall seropositive		19 (10.33, 6.33–15.66)				
Age, median (IQR)	9 (5–12) ^†^					
Age Category						
0–4 years	47 (25.5)	4 (8.51, 2.37–20.38)	Ref		Ref	
≥ 5 years	137 (74.5)	15 (10.95, 6.26 17.42)	0.74 (0.26–2.56)	0.636	0.35 (0.09–1.43)	0.14
Sex						
Male (Bulls)	101 (54.9)	14 (13.86, 7.79–22.16)	2.37 (0.89–7.21)	0.086	0.96 (0.15–13.1)	0.97
Female (Cows)	83 (45.1)	5 (6.02, 1.98–13.5)	Ref		Ref	
Location						
Babura	22 (12.0)	1 (4.55, 0.12–22.84)	Ref		Ref	
Maiadua	34 (18.5)	2 (5.88, 0.72–19.68)	1.31 (0.11–15.4)	0.829	0.58 (0.04–8.76)	0.67
Maigatari	50 (27.2)	3 (6.00, 1.25–16.55)	1.34 (0.13–13.65)	0.805	0.68 (0.08–8.25)	0.74
Sule Tankarkar	78 (42.4)	13 (16.67, 9.18–26.81)	4.2 (0.52–34.04)	0.179	3.77 (0.80–37.0)	0.1
Production system						
Extensive	158 (85.9)	17 (10.76, 6.39–16.67)	Ref		Ref	
Semi-intensive	26 (14.1)	2 (7.69, 0.95–25.13)	0.69 (0.15–3.18)	0.636	5.51 (0.65–36.2)	0.11
Usage						
Meat	23 (12.5)	2 (8.33, 1.07–28.04)	Ref		Ref	
Milk	78 (42.4)	4 (5.13, 1.41–12.61)	0.52 (0.11–2.48)	0.408	1.18 (0.18–6.15)	0.85
Labour	83 (45.1)	13 (15.66, 8.61–25.29)	**0.34 (0.10–0.97)**	**0.031**	0.21 (0.02–3.50)	0.23
Living area						
Fenced	7 (3.8)	0 (0.00, 0– 40.96)	0.612 (−1.56–5.49)	0.652	1.92 (0.07–404)	0.71
Not- fenced	177 (96.2)	19 (10.73, 6.59–16.25)	Ref		Ref	
Veterinary access						
Yes	12 (6.5)	0 (0.00, 0–40.96)	−1.156 (−6.02–0.94)	0.347	0.14 (0.00–2.91)	0.21
No	172 (93.5)	19 (11.05, 6.78–16.71)	Ref		Ref	
Water source						
Stream	163 (88.6)	18 (11.04, 6.68–16.89)	−0.116 (−2.51–4.29)	0.940	0.61 (0.02–162)	0.82
Well	18 (9.8)	1 (5.56, 0.14–27.29)	−4.510 (−3.67–4.55)	0.776	0.61 (0.01–137)	0.81
Borehole	3 (1.6)	0 (0.00, 0–70.76)	Ref		Ref	

Note: odds ratio (OR) and adjusted odds ratio (aOR).

## Data Availability

Data will be made available on reasonable request.
